# The Diabetes Treatment Satisfaction Questionnaire change version (DTSQc) evaluated in insulin glargine trials shows greater responsiveness to improvements than the original DTSQ

**DOI:** 10.1186/1477-7525-5-57

**Published:** 2007-10-10

**Authors:** Clare Bradley, Rosalind Plowright, John Stewart, John Valentine, Elke Witthaus

**Affiliations:** 1 Health Psychology Research, Dept of Psychology, Royal Holloway, University of London, UK; 2 Dept of Biostatistics, sanofi-aventis Pharma, Laval, Quebec, Canada; 3 Health Outcomes Research, Accovion GmbH, Eschborn, Germany

## Abstract

**Background:**

The results of using status measures to identify any changes in treatment satisfaction strongly suggest a need for specific change instruments designed to overcome the ceiling effects frequently observed at baseline. Status measures may leave little room to show improvement in situations where baseline ceiling effects are observed. A change version of the DTSQ (DTSQc) is compared here with the original status (now called DTSQs) version to test the instruments' comparative ability to demonstrate change.

**Methods:**

Two multinational, openlabel, randomised-controlled trials (one for patients with type 1 diabetes, the other for type 2) compared new, longer-acting insulin glargine with standard NPH basal insulin. The DTSQs was completed at baseline and the DTSQs and DTSQc at final visit by 351 English- and German-speaking patients. DTSQc scores were compared with change from baseline for the DTSQs, using 3-way analysis of variance, to examine Questionnaire, Treatment and Ceiling effects (i.e. baseline scores at/near ceiling).

**Results and discussion:**

Significant Questionnaire effects and a Questionnaire × Ceiling interaction (p < 0.001) in both trial datasets showed that the DTSQc detected more improvement in Treatment Satisfaction than the DTSQs, especially when patients had DTSQs scores at/near ceiling at baseline. Additionally, significant Treatment effects favouring insulin glargine (p < 0.001) and a Treatment × Questionnaire interaction (p < 0.019), with the DTSQc showing more benefits, were found in the type 1 trial. Results for Perceived Hyper- and Hypoglycaemia also demonstrated important differences between the questionnaires in the detection of treatment effects. Tests of effect sizes showed these differences in response to change to be significantly in favour of the DTSQc.

**Conclusion:**

The DTSQc, used in conjunction with the DTSQs, overcomes the problem of ceiling effects encountered when only the status measure is used and provides a means for new treatments to show greater value than is possible with the DTSQs alone.

## Background

Patients are inclined to make the best of their current treatment and only become aware of its drawbacks when they can compare it with something better [1]. A frequently observed feature in trials of new treatments for diabetes is therefore a relatively high level of patient satisfaction with pre-trial treatment [2-5]. This is true not only of diabetes trials. High levels of patient satisfaction are widely found across different conditions. Patient satisfaction surveys produce little variation and most respondents express positive satisfaction [6]. Researchers evaluating diabetes treatment interventions have commonly used the Diabetes Treatment Satisfaction Questionnaire in its original 'status' form (for example. [2-5]) (e.g. *How satisfied are you with your current treatment? *Response options: *very satisfied *to *very dissatisfied*). This leaves those respondents who were already very satisfied beforehand with little or no room to show improved satisfaction later in the trial [7,8]. The problem is not unique to the DTSQs. Skewed satisfaction scores reflect a real phenomenon and not a failure of the scale, so changing the scale is not a good solution. Although the skew could be dealt with statistically and the scale adjusted to fit the majority scoring pattern (i.e. skewed in favour of positive satisfaction), such an adjustment would reduce the validity of the scale for those patients who are sometimes very dissatisfied [8]. A change version of the DTSQ (DTSQc) was therefore designed to overcome such ceiling effects found with the Status version (DTSQs) [8].

Aventis (previously Hoechst Marion Roussel and now sanofi aventis) has conducted multinational Phase III trials comparing insulin glargine, a new longer-acting insulin providing constant release of insulin without a pronounced peak, with widely used NPH insulin [9-11]. Measures of psychological outcomes included the DTSQs and the new DTSQc in two languages, English and German.

It was hypothesised that the DTSQc would be more responsive to treatment change particularly when respondents had scored at or near ceiling on the DTSQs at baseline. It was also expected that treatment satisfaction would be greater with insulin glargine given its longer action and constant release of insulin without a pronounced peak, achieved with only one daily injection compared with NPH insulin which even with two injections daily provides variable release of insulin.

## Methods

### Questionnaire

The DTSQc was developed from the widely used and recommended DTSQs [e.g. [5,12,13]], available in a wide range of languages [e.g. [3,14,15]], the development of which is reported in detail elsewhere [5,16]. Both forms of the DTSQ are suitable for use by people with type 1 or type 2 diabetes. Like the DTSQs, the DTSQc contains eight items scored on 7-point scales. Six items (Qs.1 and 4–8) measure Treatment Satisfaction (dealing with: satisfaction with current treatment; convenience of the treatment; flexibility; satisfaction with own understanding of their diabetes; how likely to recommend their present treatment; and how satisfied to continue with their present treatment). These are summed to produce a total Treatment Satisfaction score. Questions 2/3, concerning Perceived Frequency of Hyperglycaemia ('Perceived Hyperglycaemia')/Perceived Frequency of Hypoglycaemia ('Perceived Hypoglycaemia') respectively, are treated separately from the satisfaction items and from each other [5,16]. On these two items, low scores represent good perceived blood glucose control. The most common factor structures seen for the DTSQs show all the Satisfaction items loading together on factor 1 (this is the only important aspect of the questionnaire structure indicating a coherent scale measuring treatment satisfaction), with the Perceived Hyper- and Hypoglycaemia items (items 2 and 3) loading together on factor 2 or separately on factors 1 and 2 or on 2 and 3. It was predicted that the same pattern would be seen with the DTSQc with all six treatment satisfaction items loading on factor 1. The wording of the items themselves is the same for both the status and change versions, the small exception being part of the wording of item 7 (recommending the treatment). The important differences lie in the wording of the response options and instructions, which, in the DTSQc, direct the respondent to compare their experience of the current treatment with their experience of treatment before the study began. DTSQs scores range from, for example, 6 = *very satisfied *to 0 = *very dissatisfied *and DTSQc scores from +3 = *much more satisfied now *to -3 = *much less satisfied now*, with 0 (midpoint), representing no change.

German was the first language version of the DTSQc to be evaluated (on an Austrian population) [17]. The original English is evaluated here (UK and S.Africa) for the first time and the German is used here for the first time in Germany and Switzerland as well as Austria.

The Status version was completed by patients at both baseline and endpoint. The Change version was completed only at endpoint. At endpoint patients completed the Status measure before completing the Change version.

### Patients

The subset of patients from the first two European trials to provide DTSQc data included: 198 patients with type 1 diabetes and 153 patients with type 2 diabetes. The two datasets were analysed separately. Of the total 351 patients in the two studies, 89 were English-speaking (N_British _= 27; N_S. African _= 62) and 262 were German-speaking (N_German _= 148; N_Austrian _= 111; N_Swiss _= 3). A principal inclusion criterion was level of glycosylated haemoglobin (GHb) at Visit 1: ≤ 12.0% for patients with type 1; 7.5-12.0% for patients with type 2 diabetes. Patients were randomised to insulin glargine or NPH insulin. During the treatment phase (type 1 study = 28 weeks; type 2 study = 52 weeks), insulin glargine was administered by subcutaneous injection once daily at bedtime; NPH human insulin was administered by subcutaneous injection once or more than once daily (depending on the previous regimen) in the type 1 study, and once only at bedtime in the type 2 study (in the type 2 study 73.6% of patients overall had not used insulin prior to study entry; in the analyses here the proportion was 75.2% with no prior experience of insulin). In the type 1 study, in addition to insulin glargine or NPH, regular human insulin was administered before each meal. Table 1 shows the whole sample, broken down by their main demographic and clinical characteristics.

**Table 1:** Demographic and clinical characteristics of the whole sample

**Characteristics**	**Type 1**		**Type 2**	
	
	**N (missing)**	**Mean (SD)**	**N (missing)**	**Mean (SD)**
Age	198 (0)	39 (12)	153 (0)	59 (10)
Sex: Male/Female	108/90 (0)	-	82/71 (0)	-
Previously on insulin: Yes/No	198/0 (0)	-	38/115 (0)	-
GHb at baseline	197 (1)	7.66 (1.13)	149 (4)	8.93 (1.12)

### Data analysis

Prior to performing psychometric analyses, the combinability of the subgroups (defined by country and type of diabetes) was tested by a method described elsewhere [18], which confirmed the acceptability of combining the samples.

#### Psychometric analysis

Psychometric analysis was carried out to check the validity and reliability of the English- and German-speaking versions (separately for language, pooled for type of diabetes), using factor analysis with principal components as the extraction method with Varimax rotation. The criterion for the number of factors extracted in the unforced analysis was the number of principal components with eigenvalues greater than 1.0. A three-factor structure was subsequently forced. Reliability analysis (including Cronbach's alpha) was conducted on the six items intended to form the Treatment Satisfaction scale.

#### Alignment of scores for comparison purposes

For the comparative responsiveness analyses between the DTSQs and DTSQc, difference scores for the DTSQs were calculated (endpoint, minus baseline scores). Thus, increases in treatment satisfaction produced positive scores and decreases negative scores. The two scales being compared have different widths. DTSQc scores range potentially from -18 to +18, while DTSQs difference scores range potentially from -36 to +36. Dividing the DTSQs difference scores by 2 provided the variable labelled DTSQsDiff, which was equivalent in range to the DTSQc scores. For Perceived Hyperglycaemia and Hypoglycaemia, positive scores indicate an increase in Perceived Hyperglycaemia or Hypoglycaemia and hence deterioration in these outcomes. Endpoint-minus-baseline DTSQs scores for these 2 items were also divided by 2 to produce DTSQsDiff scores and allow direct comparison with DTSQc scores for Perceived Hyperglycaemia and Hypoglycaemia.

Only respondents with Treatment Satisfaction scores on both DTSQsDiff and DTSQc were included; likewise with the two items concerning perceived blood glucose control.

#### Categorising scores as 'at ceiling/not at ceiling' or 'at floor/not at floor'

The terms 'at ceiling' (for Satisfaction) and 'at floor' (for Perceived Hyperglycaemia and Hypoglycaemia) would usually be defined as being just the maximum and minimum scores respectively. However, since those people with scores close to, but not at maximum/minimum would have very little room to show increases or decreases, the definitions of *At Ceiling *and *At Floor *have been widened here. *At Ceiling *Treatment Satisfaction scores are defined here as ≥ 30 (maximum = 36) and *At Floor *scores (Perceived Hyperglycaemia and Hypoglycaemia) as ≤ 1 (minimum = 0). Other scores are referred to as *Not at Ceiling *and *Not at Floor*. These categorisations were made on raw DTSQs baseline scores, prior to rescaling as described above.

#### ANOVA: comparing treatment differences shown by the two questionnaires in subgroups scoring at ceiling (floor)/not at ceiling (floor)

Analysis of responsiveness to change was carried out on the three dependent variables, change in Treatment Satisfaction, change in Perceived Hyperglycaemia, and change in Perceived Hypoglycaemia, using three-way analysis of variance (ANOVA) with Treatment (2 levels), Questionnaire (2 levels) and Ceiling effect (2 levels). Distributions were skewed for Treatment Satisfaction scores and Perceived Hypoglycaemia, so the ANOVA was carried out on raw scores and additionally on the ranks of the scores. Treatment Satisfaction scores across both questionnaires were ranked separately for each study. To lend greater meaning and clarity to the data, however, bar charts and the supporting table of results are based on raw scores. In the very few (three) instances where the ranked results differ noticeably from the raw results, these are indicated on the table.

#### Effect sizes: comparing the two questionnaires in subgroups scoring at ceiling (floor)/not at ceiling (floor)

To explore further the sensitivity to change of the questionnaires an analysis of effect sizes was conducted in addition to the analysis of variance. The effects of treatment for the two questionnaires were compared regardless of whether the treatment was NPH or glargine. Effect sizes for the two treatment satisfaction scales (DTSQsDiff and DTSQc) were compared separately for the *At Ceiling *and *Not at Ceiling *groups, by testing the mean rated change (DTSQc) or DTSQsDiff scores, by t-tests against a hypothetical mean of zero and converting the resulting t-values to values of r, as a measure of effect-size. The significance of the difference between the r values resulting from the DTSQc and DTSQsDiff measures was then tested by the use of Fisher's z transformation of r [19].

## Results

### Psychometric analyses

An unforced two-factor structure of the DTSQc was seen in both languages (see Table 2). All Treatment Satisfaction items loaded highly on Factor 1 (English range = 0.81 to 0.94; German range = 0.71 to 0.90), with the two perceived blood glucose control items loading highly on Factor 2 (English range = 0.73 to 0.81; German range = 0.74 to 0.86). When forced into three factors, the two perceived blood glucose control items separated and loaded even more highly on Factor 2 (Perceived Hyperglycaemia) and Factor 3 (Perceived Hypoglycaemia), as shown in Tables 3 and 4. Cronbach's alpha coefficient for the Treatment Satisfaction scale (i.e. excluding the two perceived blood glucose control items) was 0.92 for the English and 0.94 for the German.

**Table 2:** English and German unforced (two-factor) analyses

**Item**	**English (n = 83)**		**German (n = 250)**	
	
	**Component 1 (Satisfaction)**	**Component 2 (PercBGC)**	**Component 1 (Satisfaction)**	**Component 2 (PercBGC)**
1 Satisfied	**0.817**	-0.075	**0.878**	-0.215
2 Hyperglycaemia	-0.029	**0.807**	-0.272	**0.735**
3 Hypoglycaemia	-0.094	**0.734**	-0.103	**0.857**
4 Convenient	**0.943**	-0.149	**0.868**	-0.152
5 Flexible	**0.830**	0.076	**0.896**	-0.209
6 Understanding	**0.808**	-0.053	**0.705**	-0.249
7 Recommend	**0.812**	-0.139	**0.865**	-0.190
8 Continue	**0.915**	-0.099	**0.899**	-0.159

**Table 3:** Forced 3-factor analysis of English data (N = 83)

**Item**	**Component 1 Satisfaction**	**Component 2 Perceived Hyperglycaemia**	**Component 3 Perceived Hypoglycaemia**
1 Satisfied	**0.812**	-0.095	-0.221
2 Hyperglycaemia	-0.043	**0.968**	0.120
3 Hypoglycaemia	-0.080	0.120	**0.976**
4 Convenient	**0.944**	-0.111	-0.099
5 Flexible	**0.828**	0.124	-0.025
6 Understanding	**0.808**	-0.025	-0.051
7 Recommend	**0.818**	-0.266	0.095
8 Continue	**0.916**	-0.098	-0.037

**Table 4:** Forced 3-factor analysis of German data (N = 250)

**Item**	**Component 1 Satisfaction**	**Component 2 Perceived Hyperglycaemia**	**Component 3 Perceived Hypoglycaemia**
1 Satisfied	**0.858**	-0.320	-0.019
2 Hyperglycaemia	-0.213	**0.940**	0.192
3 Hypoglycaemia	-0.150	0.175	**0.955**
4 Convenient	**0.883**	-0.009	-0.185
5 Flexible	**0.906**	-0.088	-0.195
6 Understanding	**0.717**	-0.093	-0.242
7 Recommend	**0.854**	-0.240	-0.050
8 Continue	**0.884**	-0.247	-0.005

### Distributions of scores

As anticipated, distribution of the raw baseline DTSQs scores was skewed for Treatment Satisfaction and Perceived Hypoglycaemia [5]; while Perceived Hyperglycaemia scores were normally distributed. Those patients recording baseline Treatment Satisfaction scores *At Ceiling *accounted for nearly half (49%) of those in the type 1 trial, and almost two-thirds (62%) in the type 2 trial. For Perceived Hypoglycaemia, those scoring *At Floor *at baseline accounted for 48% (type 1) and 79% (type 2). However, only 18% and 20% of patients (for type 1 and type 2 respectively) recorded baseline Perceived Hyperglycaemia scores *At Floor*.

### ANOVA: comparing treatment differences shown by the two questionnaires in subgroups scoring at ceiling (floor)/not at ceiling (floor)

**Figure 1:**
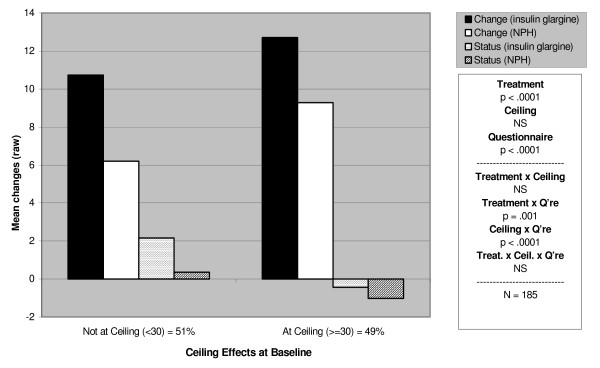
Differential responsiveness of the DTSQ change and DTSQ status: treatment satisfaction (Type 1 trial).

**Figure 2:**
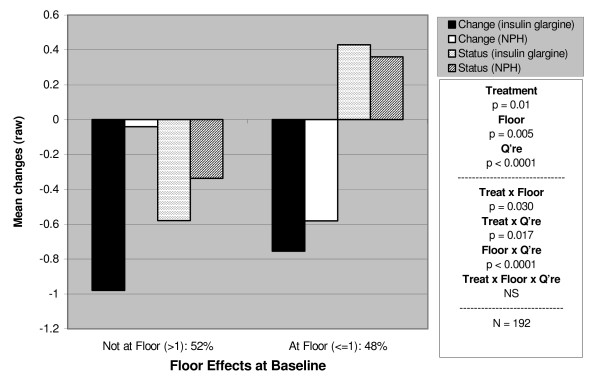
Differential responsiveness of the DTSQ change and DTSQ status: perceived frequency of hypoglycaemia (Type 1 trial).

Of the six sets of results (type 1 and type 2) for each of the three principal variables, change in Treatment Satisfaction (Tables 5 and 6), change in Perceived Hyperglycaemia (Tables 7 and 8) and change in Perceived Hypoglycaemia (Tables 9 and 10), the most relevant for comparison of the DTSQc with the DTSQs were those with skewed baseline distributions (Satisfaction and Perceived Hypoglycaemia) and attention is therefore focused primarily on these. Figures 1 and 2 provide two examples of the results, one for Treatment Satisfaction and one for Perceived Hypoglycaemia (both from the type 1 trial). For clarity of reading, all the ANOVA results, with both main effects and interactions, are presented in Tables 5, 7 and 9, and their related tables of means are given in Tables 6, 8 and 10 respectively. The text is restricted to the interpretation of these results.

**Table 5:** "Treatment Satisfaction" – Summary of Results of 3-way ANOVA (on Raw Data)

**Main Effects & Interactions**	**Type 1 Study**			**Type 2 Study**		
	
	**F**	**df1, df2**	**p**	**F**	**df1, df2**	**p**
Treatment	**15.71**	**1,181**	**<0.0001**	0.85	1,125	0.360
Ceiling	0.17	1,181	0.678	2.61	1,125	0.109*
Questionnaire	**538.04**	**1,181**	**<0.0001**	**1833.88**	**1,125**	**<0.0001**

Treatment × Ceiling	0.80	1,181	0.373	0.04	1,125	0.840
Treatment × Questionnaire	**11.70**	**1,181**	**0.001**	1.81	1,125	0.181
Ceiling × Questionnaire	**30.44**	**1,181**	**<0.0001**	**62.37**	**1,125**	**<0.0001**
Treatment × Ceiling × Questionnaire	0.00	1,181	0.948	3.18	1,125	0.077

* Ranked results were significant (*p *= 0.004)

**Table 6:** "Treatment Satisfaction" – Descriptive Statistics

	**Change Questionnaire**				**Status Questionnaire**			
	
	**glargine treatment**		**NPH treatment**		**glargine treatment**		**NPH treatment**	
	
	**At Ceiling**	**Not Ceiling**	**At Ceiling**	**Not Ceiling**	**At Ceiling**	**Not Ceiling**	**At Ceiling**	**Not Ceiling**
**Type 1 Study**								

Mean change	12.709	10.744	9.278	6.196	-0.427	2.163	-1.014	0.353
SD	7.023	4.885	6.679	7.707	2.629	3.078	2.425	3.235
*N*	*55*	*43*	*36*	*51*	*55*	*43*	*36*	*51*

**Type 2 Study**								

Mean change	16.273	14.115	15.806	14.565	4.269	0.432	2.783	0.236
SD	3.330	3.963	3.345	3.776	1.655	3.465	1.290	3.599
*N*	*44*	*26*	*36*	*23*	*44*	*26*	*36*	*23*

**Table 7:** "Perceived Hypoglycaemia" – Summary of Results of 3-way ANOVA (on Raw Data)

**Main Effects & Interactions**	**Type 1 Study**			**Type 2 Study**		
	
	**F**	**df1, df2**	**p**	**F**	**df1, df2**	**p**
Treatment	**6.86**	**1,188**	**0.010**	0.03	1,139	0.957
Floor	**8.00**	**1,188**	**0.005**	**7.45**	**1,139**	**0.007**
Questionnaire	**32.63**	**1,188**	**<0.0001**	**11.88**	**1,139**	**0.001**

Treatment × Floor	**4.80**	**1,188**	**0.030**	3.08	1,139	0.081
Treatment × Questionnaire	**5.79**	**1,188**	**0.017**^†^	1.46	1,139	0.229
Floor × Questionnaire	**65.24**	**1,188**	**<0.0001**	**23.69**	**1,139**	**<0.0001**
Treatment × Floor × Questionnaire	1.36	1,188	0.245	**6.19**	**1,139**	**0.014**

^† ^ranked results = NS (0.051)

**Table 8:** "Perceived Hypoglycaemia" – Descriptive Statistics

	**Change Questionnaire**				**Status Questionnaire**			
	
	**glargine treatment**		**NPH treatment**		**glargine treatment**		**NPH treatment**	
	
	**At Floor**	**Not Floor**	**At Floor**	**Not Floor**	**At Floor**	**Not Floor**	**At Floor**	**Not Floor**
**Type 1 Study**								

Mean change	-0.755	-0.980	-0.581	-0.041	0.429	-0.578	0.360	-0.337
SD	1.422	1.378	1.277	1.471	0.520	0.586	0.630	0.860
*N*	*49*	*51*	*43*	*49*	*49*	*51*	*43*	*49*

**Type 2 Study**								

Mean change	-0.842	-1.350	-1.393	-0.300	0.395	-0.950	0.295	-1.200
SD	1.645	1.599	1.510	1.947	0.854	0.742	0.666	0.823
N	*57*	*20*	*56*	*10*	*57*	*20*	*56*	*10*

**Table 9:** "Perceived Hyperglycaemia" – Summary of Results of 3-way ANOVA (on Raw Data)

**Main Effects & Interactions**	**Type 1 Study**			**Type 2 Study**		
	
	**F**	**df1, df2**	**p**	**F**	**df1, df2**	**p**
Treatment	0.89	1,185	0.346	0.04	1,137	0.841
Floor	1.01	1,185	0.315	1.51	1,137	0.221
Questionnaire	**28.72**	**1,185**	**<0.0001**	**8.88**	**1,137**	**0.003**

Treatment × Floor	3.46	1,185	0.064	1.19	1,137	0.278
Treatment × Questionnaire	0.34	1,185	0.558	0.02	1,137	0.901
Floor × Questionnaire	**35.08**	**1,185**	**<0.0001**	**37.55**	**1,137**	**<0.0001**
Treatment × Floor × Questionnaire	**3.93**	**1,185**	**0.049**^‡^	0.94	1,137	0.335

^‡ ^ranked results = NS (0.067)

**Table 10:** "Perceived Hyperglycaemia" – Descriptive Statistics

	**Change Questionnaire**				**Status Questionnaire**			
	
	**glargine treatment**		**NPH treatment**		**glargine treatment**		**NPH treatment**	
	
	**At Floor**	**Not Floor**	**At Floor**	**Not Floor**	**At Floor**	**Not Floor**	**At Floor**	**Not Floor**
**Type 1 Study**								

Mean change	-1.208	-0.767	-1.600	0.171	0.313	-0.459	0.350	-0.311
SD	1.414	1.603	1.430	1.530	0.673	0.798	0.669	0.706
*N*	*24*	*73*	*10*	*82*	*24*	*73*	*10*	*82*

**Type 2 Study**								

Mean change	-0.933	-0.644	-1.462	-0.185	0.600	-1.025	0.385	-0.972
SD	1.831	2.057	2.184	2.066	0.967	1.112	0.961	0.949
*N*	*15*	*59*	*13*	*54*	*15*	*59*	*13*	*54*

### Change in treatment satisfaction

#### Type 1 trial

Treatment Satisfaction increased overall in both the insulin glargine and NPH treatment groups, but with a significantly greater increase in the insulin glargine group. Figure 1 shows the DTSQc was markedly more responsive than the DTSQsDiff across all subgroups, including the *Not at Ceiling *group. The strong Ceiling × Questionnaire interaction indicates that, among those patients with baseline scores *At Ceiling*, the DTSQc showed increases in Treatment Satisfaction, while the DTSQsDiff showed small mean decreases. Using the DTSQc, the *At Ceiling *group showed a greater increase in Satisfaction than the *Not at Ceiling *group. However, it was expected that the *Not at Ceiling *group would be better able to show an increase in Treatment Satisfaction than the *At Ceiling *group, if they were using the DTSQsDiff, and this was indeed the case. As Figure 1 also shows, no matter how patients scored at baseline, the insulin glargine group showed greater improvement in Treatment Satisfaction than the NPH group when scoring with the DTSQc than when scoring with the DTSQsDiff (see Table 5, treatment × questionnaire interaction).

#### Type 2 trial

Unlike the type 1 study, no main effect of Treatment was seen for patients with type 2 diabetes. However, as in the type 1 trial, the DTSQc detected greater increases in satisfaction than the DTSQsDiff, and the interaction with Ceiling effects shows that once again the difference between status and change versions of the DTSQ was especially marked in the *At Ceiling *subgroup (see Table 6).

### Change in perceived hypoglycaemia

#### Type 1 trial

Patients with type 1 diabetes showed an overall reduction in Perceived Hypoglycaemia, with those in the insulin glargine group showing a significantly greater reduction. There was a main effect of Questionnaire, with the DTSQc showing greater effects (see Figure 2). Reductions in Perceived Hypoglycaemia were observed in all the Questionnaire × Floor/Not at Floor subgroups except one: when those *At Floor *were scoring on the DTSQsDiff, they showed a slight increase. The reductions in the other subgroups were seen most clearly among those *At Floor *using the DTSQc (see Table 7, the significant Floor × Questionnaire interaction). The DTSQc also showed a greater responsiveness than the DTSQsDiff to Treatment differences (Questionnaire × Treatment interaction), although this did not quite reach significance on the ranked (two-tailed) test (p = 0.051).

#### Type 2 trial

Treatment effects on Perceived Hypoglycaemia observed in the type 2 participants only became significant in the interaction with Questionnaire and Floor effect. Regardless of Treatment, there were greater reductions overall in Perceived Hypoglycaemia in those *Not at Floor *to start with (main effect of floor). The DTSQc showed more improvements in Perceived Hypoglycaemia than the DTSQsDiff (main effect of Questionnaire), principally because the change version continued to detect improvements where there were Floor effects, whereas the status version did not (see the highly significant Floor × Questionnaire interaction in Table 7). When using the status measure with people *At Floor*, an increase in Perceived Hypoglycaemia is apparent, while the change version shows reductions (i.e. improvements) in Perceived Hypoglycaemia for both insulin glargine and NPH.

### Change in perceived hyperglycaemia

#### Type 1 trial

Only 18% of people fell into the *At Floor *subgroup on this variable. However, the principal features observed with the Treatment Satisfaction results, such as the main effect of Questionnaire and the interaction between Questionnaire and Floor effect, were observed once again, with the Change version showing greater improvements (reductions) in Perceived Hyperglycaemia than the Status version, particularly in those already reporting little hyperglycaemia at baseline.

#### Type 2 trial

Only 20% of people fell into the *At Floor *subgroup. The DTSQc showed significantly greater improvements in Perceived Hyperglycaemia than the DTSQs (Questionnaire effect) in those people reporting little hyperglycaemia at baseline (interaction with Floor effect). However, as in the Type 1 trial, in the people *At Floor*, Perceived Hyperglycaemia was seen to increase in frequency when people were scoring on the DTSQsDiff.

### Effect sizes: comparing the responsiveness to treatment differences of the two questionnaires in subgroups scoring at ceiling (floor)/not at ceiling (floor)

In the type 1 study, for the *Not at Ceiling *group, the t-value for the DTSQsDiff Treatment Satisfaction measure was t(94) = 3.30 and for the DTSQc was t(97) = 11.97, which translate into r values of 0.32 and 0.77, respectively. The z score for the difference between the Fisher transformation of these values is z = 4.73, indicating a markedly larger effect size for the DTSQc than for the DTSQsDiff (see Table 11). For the *At Ceiling *group the corresponding values were t(91) = 2.44, r = 0.25 for the DTSQsDiff and t(95) = 15.64, r(change) = 0.85 for the DTSQc, and the z score of the difference between these r values = 10.15, an even more marked difference than for the *Not at Ceiling *group. Each of these t-tests is for independent groups. Since the two measures (DTSQsDiff and DTSQc) are not independent, these two t-tests are not independent of each other. Thus, for example, in the type 1 study for the treatment satisfaction variable in the glargine group Spearman's rho is 0.36, n = 99, p < 0.001 for the correlation between DTSQsDiff and DTSQc. Given these positive correlations it is more difficult to show differences between the two questionnaires. However, as the effect sizes based on these ts differ significantly despite the positive correlations, the stated results are conservative. For both groups, *At Ceiling (Floor)/Not at Ceiling (Floor)*, therefore, the change measure is significantly more responsive than the DTSQsDiff measure. Furthermore, a simple test of the difference between these two z scores (4.73 and 10.15), using a standard error of the square root of 2, gives a final z value of 3.83, indicating that the superior responsiveness of the change measure is significantly greater in the *At Ceiling *group than in the *Not at Ceiling *group. Equivalent figures for the Perceived Hypo/Hyperglycaemia variables and for the type 2 trial follow a similarly significant pattern (see Table 11).

**Table 11:** Comparison of effect sizes for differences between the DTSQc and DTSQsDiff scores

** Study**	**At Ceiling (("AC")/ At Floor ("AF")**	**t (df)**		**D (= Z of difference between t for DTSQc and t for DTSQsDiff)**	**Z of difference between D in the AC/Not AC groups or in the AF/Not AF groups**
				
		**DTSQc Questionnaire**	**DTSQsDiff Questionnaire**		
**Treatment Satisfaction**					

Type 1	AC	15.64 (95)	-2.44 (91)	10.15	3.83****
	Not AC	11.97 (97)	3.30 (94)	4.73	

Type 2	AC	27.10 (89)	0.39 (83)	11.23	4.05****
	Not AC	26.81 (52)	7.37 (50)	5.51	

**Perceived Hypoglycaemia**					

Type 1	AF	-4.73 (95)	6.65 (91)	-7.54	-6.71****
	Not AF	-3.48 (99)	-6.62 (100)	1.94	

Type 2	AF	-7.37 (114)	4.79 (113)	-8.07	-7.47****
	Not AF	-2.70 (32)	-7.40 (29)	2.49	

**Perceived Hyperglycaemia**					

Type 1	AF	-5.48 (33)	2.85 (33)	-5.22	-5.73****
	Not AF	-2.14 (159)	-6.45 (156)	2.88	

Type 2	AF	-3.15 (27)	2.77 (28)	-3.84	-4.08****
	Not AF	-2.27 (113)	-10.32 (116)	1.93	

(**** <0.0001)

Using non-parametric tests on the same Type 1 treatment satisfaction data, a similarly high and significant final z value of 3.17 was obtained. Similar patterns of results, showing that the superiority of the change measure was significantly greater when patients scored at or near ceiling at baseline, were obtained for treatment satisfaction in the Type 2 study. (Since the non-parametric results are similarly significant, only the parametric results are presented in Table 11).

The pattern of results for the perceived frequency of hyper- and hypoglycaemia also showed markedly greater responsiveness of the DTSQc measure (compared with the DTSQsDiff) in the *At Floor *groups. Although Table 11 suggests that the DTSQsDiff may be more responsive to (either) treatment than the DTSQc in the *Not at Floor *group, the advantage is small compared with the advantage of the DTSQc in the *At Floor *group and inspection of Figure 2 reveals that the DTSQc showed bigger differences between the two treatments studied, glargine and NPH. As the non-parametric tests again mirrored the parametric tests, only the latter are presented.

## Discussion

The expected structure and reliability of the DTSQc is confirmed in both languages. Howorka and colleagues [17] have already found the German DTSQc to perform well with Austrian patients with type 1 diabetes comparing meal-related insulins. The insulin glargine studies here add to that work, providing validation of the English version (for UK and S.Africa), confirming the validity of the German version for Austria and now Germany for the first time, for patients with both type 1 and type 2 diabetes. Additionally, the impact of ceiling and floor effects is investigated here in detail.

Given the nature of insulin glargine, with its longer action and constant release of insulin without a pronounced peak, achieved with only one daily injection of basal insulin, it was expected that treatment satisfaction would be greater in the insulin glargine group than the NPH group. Once-daily insulin glargine has been shown to be associated with at least similar glycaemic control with fewer hypoglycaemic episodes than NPH insulin [9,20,21]. The results reported here show significant benefits in Treatment Satisfaction and Perceived Hypoglycaemia from using insulin glargine among patients with type 1 diabetes. Benefits of insulin glargine are also seen among patients with type 2 diabetes, although these only show up in the three-way interaction of treatment × questionnaire × floor effect for Perceived Hypoglycaemia and not in the main effects of Treatment. Thus benefits attributable to glargine, which would not be revealed by the DTSQs alone, are revealed by the DTSQc when used with people scoring at or near floor at baseline. Treatment effects on the DTSQs results in the datasets from other countries included in these Phase III trials are reported elsewhere [10,11].

It was predicted that the DTSQc would prove to be more responsive to change than the DTSQs and the present results confirm this. The differences expected were principally between the two Questionnaires and their interaction with Ceiling/Floor effects and these were found to be highly significant for all three variables (measuring changes in Treatment Satisfaction, Perceived Hyperglycaemia and Hypoglycaemia) across both trials (type 1 and 2). The DTSQc performed as intended, showing significantly enhanced responsiveness to improvements in Treatment Satisfaction, particularly among patients *At Ceiling *at baseline, as well as greater responsiveness to improvements in perceived blood glucose control. In those patients with baseline scores *At Ceiling/Floor*, the DTSQc consistently showed improvements with glargine across all variables and both patient populations (i.e. type 1 and 2). In contrast, among those *At Ceiling/Floor*, the DTSQsDiff showed either a much smaller improvement, or more often in the *At Ceiling/Floor *groups, a slight deterioration. In the *At Ceiling/Floor *groups, those few patients reporting decreased satisfaction or increased hypo- or hyperglycaemia have a disproportionate influence on mean scores of the DTSQsDiff, as patients wishing to register improvements had much less scope for doing so than those wishing to register a deterioration. The new Change measure allowed patients equal opportunity for indicating improvement and deterioration. As expected, which questionnaire was used mattered less for the *Not at Ceiling *group, since patients still had room to show increases in Satisfaction when using the DTSQs, and did so.

The breakdown of the data into subgroups of participants *At Ceiling/Floor *and those *Not at Ceiling/Floor *is necessarily an imperfect division, based as it is on splitting scores along a continuum. Nevertheless, the division has served in these analyses to highlight clearly the limitations that can be associated with measuring change in satisfaction using a status measure, where so many people, who have already scored at or near ceiling at baseline, wish to register marked improvements at endpoint; likewise for those recording optimal baseline scores at or near floor for Perceived Frequency of Hyperglycaemia or Hypoglycaemia. Thus the division has made it possible to have a clearer picture of the benefits of using an explicit change measure at endpoint.

It is important to note that, although the benefits of the change measure are most apparent in the Treatment Satisfaction scores from patients scoring *At Ceiling *at baseline, the DTSQc is also more responsive to change than the DTSQsDiff in patients who were *Not at Ceiling *to start with. Figure 1, for example shows that mean improvements in satisfaction (using the Change measure) of 10.74 were shown in the *Not at Ceiling *insulin glargine group. Patients would have to score 25 or less (out of a possible 36) on the DTSQs at baseline to be able to register such an improvement in DTSQs scores and few do score this low to start with. Thus, even in the *Not at Ceiling *subgroups, defined here as scoring <30 on the DTSQs at baseline, ceiling is reached by many who wish to register marked improvements in satisfaction. The DTSQc shows enhanced responsiveness to change in these patients too. In the case of the two perceived blood glucose control items, the DTSQc is unequivocally more responsive to improvements in hypoglycaemia for those in the *At Floor *group. For those *Not at Floor*, the DTSQsDiff measure shows greater responsiveness to change as indicated by the D values shown in Table 11. However the advantages of the DTSQc measure in the *At Floor *group far outweigh the advantages of the DTSQsDiff measure in the *Not at Floor *group. Furthermore the effect size measures reported in Table 11 do not refer to differences between the insulin treatments but rather refer to change associated with either treatment. For indications of responsiveness to differences between glargine and NPH we can refer to Figure 2 which suggests that the DTSQc is more responsive to change associated with insulin glargine (the newly introduced treatment in this Type 1 trial) than is the DTSQsDiff measure particularly in the *Not at Floor *group. As expected in this Type 1 trial illustrated in Figure 2, there were less marked changes from baseline for the NPH treatment group, most of whom would have used NPH insulin prior to baseline as well as during the study. Therefore, it remains the case that for the Treatment Satisfaction variable and for the Perceived Frequency of Hyper- and Hypoglycaemia variables, the DTSQc is the measure of choice, whether or not patients score at/near ceiling at baseline.

Other authors have compared prospective (status) and retrospective (change) measures of health status and symptoms and, as in the present dataset, found more pronounced changes with the retrospective change measure following treatment, than with the status measure used pre- and post- treatment [22]. Aseltine and colleagues suggested that status measures may not be an error-free method of measuring change, because patients' standards, or the criteria they use to determine their ratings, may change following treatment as their frame of reference may change, so that apparently identical ratings are not, in fact, comparable. Aseltine et al did not consider the role of ceiling or floor effects in limiting the validity of status measures, although the data they presented suggest that ceiling effects with the status version of the health status measure they used may have increased the difference between the status and change measures, while the symptom measure used appeared little affected by ceiling or floor effects and also gave rise to fewer discrepancies between status and change measures. Where the capacity for change in status scores is limited by ceiling or floor effects, a retrospective change measure with no such limitations will provide a more valid measure of the benefits of treatment.

Another potential concern with a retrospective measure of change is that it might reflect a socially desirable tendency to report improved satisfaction whatever the treatment rather than genuine change. The evidence is that the DTSQc does not. Howorka et al [17] found in a crossover study that patients who went back in the second phase from lispro to standard soluble insulin showed a decline in satisfaction on the retrospective measure, indicating that the DTSQc does not always invoke positive responding. Moreover the DTSQc was used in a recent waiting list control trial [23]: although only the DTSQs results were included in the 2002 BMJ paper [23], the DTSQc was used pre- and post-intervention in waiting list controls and showed no change immediately pre-intervention (compared with baseline), but marked changes post-intervention (Speight, personal communication 2003). These studies suggest that the DTSQc does not simply elicit positive change responses regardless of treatment circumstances, but does genuinely reflect a response to improvements in treatment borne out by experience.

The DTSQc is designed specifically for use in a treatment intervention situation to allow explicit retrospective comparison between treatments. In studies of treatment interventions, where patients' baseline scores show no ceiling effect, researchers will not necessarily need to use the DTSQc at endpoint. Since the DTSQc is a measure of comparative satisfaction, a measure of absolute Satisfaction should always be used at baseline using the original Status version of the DTSQ, to enable researchers to put the findings of the Change measure into context, i.e. the DTSQc alone will not indicate how satisfied or dissatisfied the person was in the first place. Researchers may wish to include the Status version at endpoint as well (prior to completion of the Change version), in view of the apparent greater responsiveness of the DTSQsDiff measure in these studies for those with poorer perceived blood glucose control at baseline (i.e. *Not at Floor)*. This would also enable them to draw comparisons between their findings and those from earlier studies using only the DTSQs.

A further possible advantage of retrospective change measures is that they may correspond more closely to the kind of reports that patients are most likely to give in routine clinical practice. Thus research findings based on retrospective change measures are likely to translate more directly into clinical practice. The one study of insulin lispro that used the DTSQ Change measure was able to show the substantial increases in patient satisfaction [17] apparent to clinicians in their routine practice, instead of the more modest, though significant, improvements apparent with the DTSQ Status [17].

## Conclusion

Although the DTSQc measure was introduced to combat problems resulting from ceiling effects, it was found here that the measure of treatment satisfaction derived from the DTSQc questionnaire was more responsive than that derived from the DTSQsDiff measure, even when scores were not at ceiling, and so can be recommended as the method of choice for measuring change in satisfaction alongside the DTSQs.

Using a status measure, patients' satisfaction with a new treatment may appear to be no different from their satisfaction with their old treatment when they already felt satisfied with the previous treatment, and differences between treatments may be underestimated. This can give rise to potentially misleading conclusions, as patients appear not to value a new treatment as highly as in fact they do. In these insulin glargine studies the change version of the DTSQ has shown that it is able to overcome the effects of baseline ceiling/floor scores on outcomes. It enables patients to record their experience of change in satisfaction and perceived blood glucose control more precisely and thus provides greater validity in determining the benefits or drawbacks of a new treatment.

## Authors' contributions

CB designed and developed the DTSQs and DTSQc, advised HMR/Aventis/sanofi aventis on the use of the questionnaires in the glargine trials, analysis and interpretation, and led the writing of the paper.

RP contributed to the translation work on the DTSQs and DTSQc, did part of the statistical analyses and an outline draft of the paper.

JS conducted the analyses of the DTSQ data, contributed to reports and papers on the glargine trials, advised on part of these statistical analyses and contributed to the discussion of the responsiveness analyses.

JV carried out analyses of the effect sizes, and contributed to the interpretation and to manuscript preparation.

EW was responsible for guiding the assessment of patient reported outcomes in the glargine trials, for reporting and publishing findings from these trials, and contributed to the discussion of these results and preparation of this manuscript.

All authors approved the final manuscript
